# The use of geosocial networking smartphone applications and the risk of sexually transmitted infections among men who have sex with men: a systematic review and meta-analysis

**DOI:** 10.1186/s12889-018-6092-3

**Published:** 2018-10-16

**Authors:** Haidong Wang, Lu Zhang, Ying Zhou, Keke Wang, Xiaoya Zhang, Jianhui Wu, Guoli Wang

**Affiliations:** School of Public Health, North China University of Science and Technology, No. 21 Bohai Road, Tangshan Bay Eco City, Caofeidian District, Tangshan, 063210 People’s Republic of China

**Keywords:** Geosocial networking application, App, MSM, HIV, Sexually transmitted infection

## Abstract

**Background:**

Geosocial networking smartphone applications (apps) are popular tools for seeking sexual partners among men who have sex with men (MSM). We evaluated app use and risk of sexually transmitted infections (STIs) in app-using MSM (app-users) by a systematic review and meta-analysis.

**Methods:**

A literature search for relevant studies was performed. We extracted date of STIs (ever being diagnosed with human immunodeficiency virus [HIV], syphilis, gonorrhea and chlamydia) and sexual behavior (e.g., number of app-met partners, unprotected anal/oral sex, HIV testing) from the eligible studies. Pooled proportions and odds ratios (ORs) with 95% confidence intervals (95% CIs) were estimated.

**Results:**

Twenty-five studies were included. The self-reported prevalence of prior diagnosis of HIV among app-users ranged from 2.2 to 37.7%, and the pooled prevalence of HIV infection was 6% (95% CI, 4–11%). Compared with non-users, app-users were more likely to have gonorrhea (OR = 2.36; 95% CI, 2.07–2.70) and chlamydia (OR = 2.22; 95% CI, 1.92–2.56). The two groups were similar in terms of diagnoses of HIV (OR = 0.89, 95% CI, 0.68–1.16) and syphilis (OR = 1.92; 95% CI, 0.91–4.03). However, when one study that caused substantial heterogeneity was omitted, the pooled OR for app-users to contract syphilis became 3.00 (95% CI, 1.84–4.91) .

**Conclusions:**

MSM who seek sexual partners using apps may be more likely to have STIs as than are non-users.

**Electronic supplementary material:**

The online version of this article (10.1186/s12889-018-6092-3) contains supplementary material, which is available to authorized users.

## Background

The prevalence of men who have sex with men (MSM)-related human immunodeficiency virus (HIV) infection is increasing worldwide [1, 2]. Advances in communication technology now offer MSM different opportunities to meet sexual partners. In recent years, a number of global positioning system (GPS)-equipped smart phone applications (geosocial networking smartphone applications; apps) have been developed (e.g., Jack’d, Scruff, Blued, and Grindr) that are popular tools in the MSM community [3]. These apps allow subscribers to create individualized profiles, share photos, and send their location. Users can also send instant messages to other users who are in close (or least identified) proximity, effectively allowing MSM to arrange sexual encounters. From 2009 to 2013, these apps have been used increasingly among MSM. Approximately 40% of MSM reported using these apps to seek sex partners in 2013 [4]. The first of these apps, Grindr (launched in 2009), reported it had reached approximately 6 million users around the world in 2013, with an estimated 8000 new users every day [3, 5].

With the proliferation of apps, increased use of these apps may facilitate finding casual sexual partners, resulting in unsafe sexual practices [6]. Prior work has shown that MSM who use these apps (app-users) tend to have more sexual encounters, more frequent anal intercourse, more unprotected sex, and a larger number of sexual partners known to have HIV and other STIs [7–10]. This increases their risk for HIV and STIs acquisition/transmission, compared with MSM who used different channels to seek sex partners (non-users) [11, 12]. However, evidence among these studies is inconsistent. Some studies suggested that app-users may be more likely to practice safer sex with these partners than are non-users [5, 13], and that use of apps was not associated with increased risky behavior for STIs transmission [14]. Therefore, a comprehensive summary of apps usage and their associated effects on sexual health is warranted.

The aims of the present study were to: (1) examine the characteristics of app-users; (2) summarize the existing evidence on the use of apps and associated sexual behaviors among app-users; and (3) compare STIs diagnoses in apps-users with those of non-users.

## Methods

### Literature search

This meta-analysis report followed the guidelines of Preferred Reporting Items for Systematic Reviews and Meta-analysis (PRISMA) [15]. We identified relevant articles published up to 9th October, 2017 by a systematic search of MEDLINE via PubMed, using the key words “homosexual”, “gay”, “bisexual”, “men who have sex with men”, “MSM”, “applications”, “apps”, “phone”, “smartphone”, “mobile phone”, “cellphone”, “Grindr”, “Jack’d”, “Scruff”, “Hornet”, “Blued”, “SpaceFinder”, “GSN”. In order to identify additional potentially relevant articles, the reference lists of included articles were manually searched by researchers.

### Study selection

All articles that reported use of apps and their associated effects on sexual health and/or sexual behaviors among MSM were assessed. We selected articles adhering to the following criteria: (1) focus on app-using MSM or studies involving both app-users and non-users; (2) reported data for sexual health or sexual behaviors; and (3) full texts were available. Only English-language studies were considered.

Exclusion criteria were as follows: (1) studies that reported non-users only; and (2) studies that lacked required data on outcomes of interest.

Three authors (HW, LZ, YZ) independently assessed the retrieved records. The study selection process was conducted in two steps: first, titles and abstracts were analyzed and preselected according to inclusion and exclusion criteria; second, full texts of potentially eligible articles were retrieved for further evaluation. Disagreements were resolved by consensus.

### Data extraction

Three authors (HW, KW, XZ) independently reviewed the full text of eligible studies and extracted the following information: (1) study details: first author, year of publication, study location, study period, recruitment method, main study objective; (2) characteristics of the study population: age, sexual orientation, education, race/ethnicity; and (3) outcomes of interest: HIV/STIs diagnoses (ever being diagnosed with HIV, syphilis, gonorrhea and chlamydia) and app related sexual behaviors (e.g., number of sex partners found through the platform). Disagreements were resolved by consensus.

### Statistical analysis

Meta-analysis was performed using R software with the Meta package (version 3.2.0). The Higgins I^2^ statistic was used to test for heterogeneity among studies, with I^2^ < 25% considered low heterogeneity, 25–75% considered medium heterogeneity, and > 75% considered high heterogeneity [16]. If middle or high heterogeneity existed among studies, a DerSimonian-Laird random-effects model was used to calculate pooled proportion or odds ratios (ORs) and corresponding 95% confidence intervals (95% CIs) [17]. A Mantel-Haenszel fixed-effects model was used in the absence of heterogeneity [18, 19]. If there was heterogeneity, we performed sensitivity analysis to test the reliability of the results. In addition, Begg’s and Egger’s tests were used to evaluate publication bias, with *P* > 0.05 indicating no publication bias [20].

## Results

### Characteristics of included studies

**Fig. 1:**
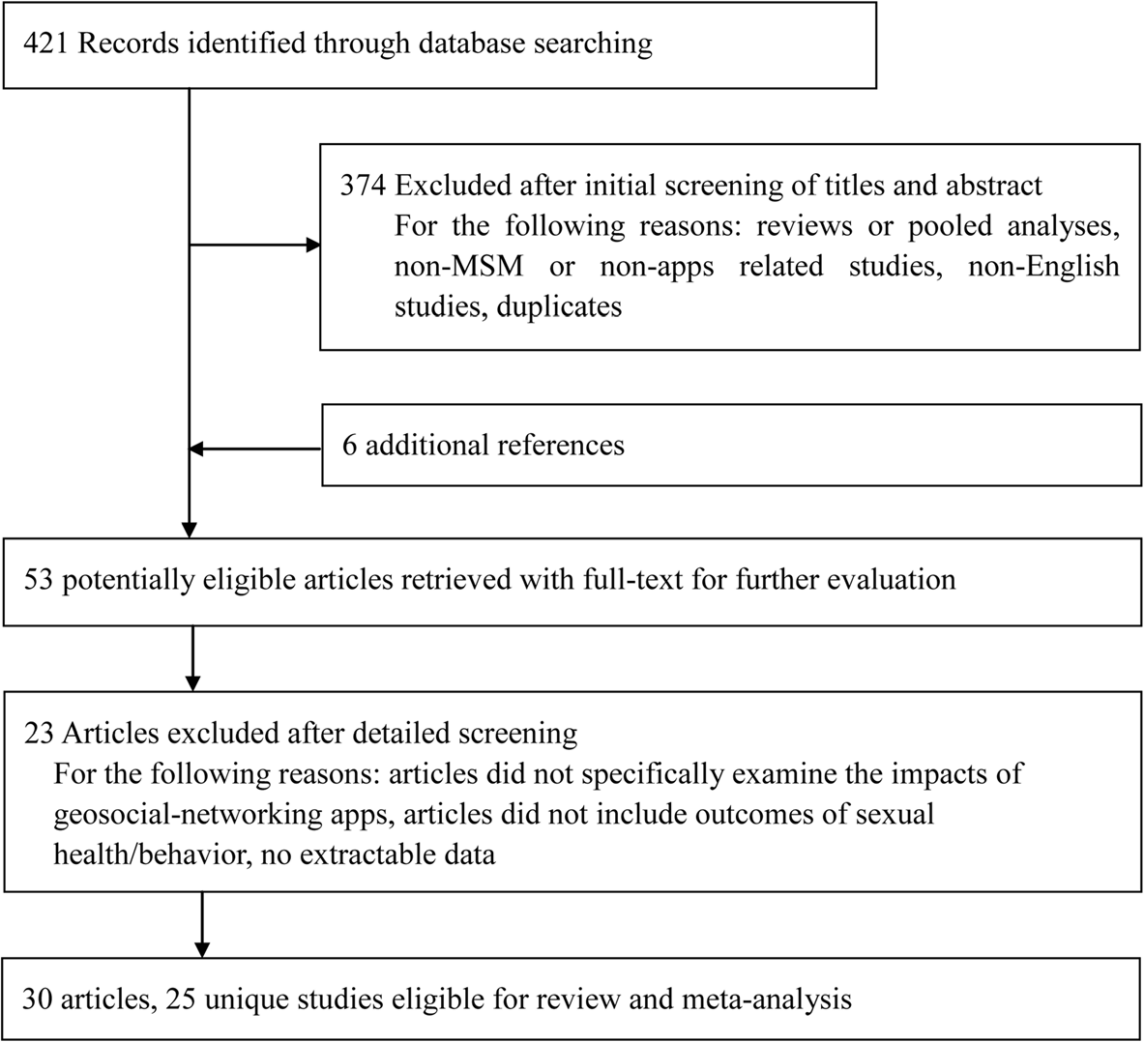
Flow diagram of the study selection process

A total of 421 articles were identified from the database search; six additional papers was found through a reference check; 53 potentially eligible articles were retrieved for more detailed analysis. Ultimately, 25 studies were included for the meta-analysis [3–6, 13, 21–45]. The flow diagram of the study selection process is shown in Fig. 1.

The characteristics of the included studies are summarized in Additional file 1: Table S1. All studies were cross-sectional; 17 were conducted in the United States, five in China, and one each in Australia, Thailand and India. Ten studies recruited MSM through apps [5, 21, 22, 26, 30, 34, 36, 38, 42, 43]. Other studies applied a variety of recruitment methods, including gay websites, fixed venues, and social service organizations serving MSM. Data collection year of MSM ranged from 2009 to 2015. Most studies (*n* = 20, 80.0%) evaluated sexual behaviors/characteristics of app-users [3–6, 13, 21–23, 26–31, 33, 35–42].

### Demographic characteristics

More than half of these studies (*n* = 15/25, 60.0%) recruited app-users aged were 18 or above years of age [5, 13, 21–27, 29, 32, 34, 36–38, 41, 43–45], and showed a predominance of young adults (18 to 30 years old; *n* = 8/15, 53%) [5, 13, 21, 22, 25, 36, 38, 41, 43–45]. According to the available data, 4427 (54.2%) app-users were white, 5754 (78.2%) were gay-identified and 6420 (71.3%) had at least college education. 1748 (71.3%) non-users were white, 2575 (47.9%) were gay-identified and 5791 (74.3%) had at least college education (Table 1).

**Table 1:** Demographic characteristics of app users and non-app users

First author (Year)	Age Mean ± SD or n (%)			Sexual orientation n (%)			Race/ethnicity n (%)			Education n (%)		
	Group	app users	non-app users	Group	app users	non-app users	Group	app users	non-app users	Group	app users	non-app users
Goedel (2015)	18–66	31.73 ± 10.7	NR	Gay	71 (77.2)	NR	White	58 (63.0)	NR	<College	45 (48.9)	NR
				Other	21 (22.8)		Other	34 (37.0)		≥College	47 (51.1)	
Goedel (2016) Duncan (2016)	18–30 ≥31	98 (56.6)	NR	Gay	146 (84.9)	NR	White	69 (39.9)	NR	<College	96 (55.5)	NR
		75 (43.4)		Other	26 (15.1)		Other	104 (60.1)		≥College	77 (44.5)	
Phillips (2014)	18–34	160 (66.4)	70 (50.7)	Gay	220 (91.7)	111 (81.6)	White	120 (49.8)	60 (43.5)	<College	44 (18.3)	22 (15.9)
	≥35	81 (33.6)	68 (49.3)	Other	20 (8.3)	25 (18.4)	Other	121 (50.2)	78 (56.5)	≥College	197 (81.7)	116 (84.1)
Rhoton (2016)	≥18	29.46 ± 8.20	NR	Gay	2 (0.4)	NR	NR			<College	384 (87.0)	NR
				Other	406 (90.4)					≥College	57 (12.0)	
Holloway (2015) Holloway (2015)	≥25	30.66 ± 6.68	NR	Gay	265 (90.1)	NR	White	152 (51.5)	NR	<College	33 (11.2)	NR
				Other	29 (9.9)		Other	143 (48.5)		≥College	262 (88.8)	
Ko (2016)	18–54	27.3 ± 6.8	26.5 ± 6.6	NR			NR			<College	69 (17.3)	120 (18.2)
										≥College	331 (82.7)	540 (81.8)
Beymer (2014)	≤29	1287 (49.7)	1823 (27.9)	NR			White	1366 (52.8)	2198 (47.8)	<College	287 (11.1)	753 (16.4)
	≥30	1302 (50.3)	2772 (72.1)				Other	1223 (47.2)	2397 (52.2)	≥College	2302 (88.9)	3842 (83.6)
Beymer (2016)	NR			NR			White	109 (74.7)	NR	<College	17 (11.6)	NR
							Other	37 (25.3)		≥College	129 (88.4)	
Yeo (2016)	17–26	21.52 ± 2.29	NR	Gay	159 (74.6)	NR	Chinese	206 (96.7)	NR	<College	47 (22.2)	NR
				Other	54 (25.4)		Other	7 (3.3)		≥College	165 (77.8)	
Winetrobe (2014) Rice (2012)	18–24	21.8 ± 1.7	NR	Gay	168 (86.2)	NR	White	76 (39.0)	NR	<College	30 (15.4)	NR
				Other	27 (13.8)		Other	119 (61.0)		≥College	165 (84.6)	
Tang (2016)	≤29	680 (82.5)	424 (70.7)	Gay	626 (76.0)	412 (68.7)	NR			<College	186 (22.6)	183 (30.5)
	≥30	144 (17.5)	176 (29.3)	Other	198 (24.0)	188 (31.3)				≥College	638 (77.4)	417 (69.5)
Muessig (2013) LeGrand (2014)	18–30	24 ± 3.0	NR	NR			Black	22 (100)	NR	NR		
Chow (2016) Chow (2017)	NR			NR			NR			NR		
Allen (2017)	18–29 ≥30	65 (34.6)	212 (37.8)	Gay	164 (87.2)	434 (77.4)	Black	86 (45.7)	270 (48.1)	<College	125 (66.5)	389 (69.3)
		123 (65.4)	349 (62.2)	Other	24 (12.8)	127 (22.6)	Hispanic	102 (54.3)	291 (51.9)	≥College	63 (33.5)	172 (30.7)
Bien (2015)	16–25 ≥26	156 (28.6)	161 (20.2)	Gay	428 (78.7)	543 (69.1)	NR			<College	224 (41.5)	396 (50.1)
		389 (71.4)	636 (79.8)	Other	116 (21.3)	243 (30.9)				≥College	316 (58.5)	395 (49.9)
Rendina (2014)	≥18	30.1 ± 9.1	NR	Gay	1162 (86.0)	NR	White	666 (49.3)	NR	NR		
				Other	189 (14.0)		Other	685 (50.7)				
Grosskopf (2014)	NR	Mdn 24.83	Mdn 27.75	NR			White	30 (76.9)	47 (54)	<College	5 (13.9)	15 (21.7)
							Other	9 (23.1)	40 (46)	≥College	31 (86.1)	54 (78.3)
Goedel (2016)	18–30 ≥31	94 (62.7)	NR	Gay	126 (84.0)	NR	White	66 (44.0)	NR	<College	64 (42.7)	NR
		56 (37.3)		Other	24 (16.0)		Other	84 (56.0)		≥College	86 (57.3)	
Lehmiller (2014)	NR	30.7 ± 10.1	28.9 ± 11.7	Gay	(86.9)	(73.1)	White	(86.7)	(86.0)	NR		
				Other	(13.1)	(26.9)	Other	(13.3)	(14.0)			
Goedel (2017)	18–30 ≥31	78 (38.6)	NR	Gay	176 (87.1)	NR	White	143 (72.2)	NR	NR		
		124 (61.4)		Other	26 (12.9)		Other	55 (27.8)				
Landovitz (2013)	18–29 ≥30	349 (93.1)	NR	NR			White	159 (42.4)	NR	NR		
		26 (6.9)					Other	216 (57.6)				
Burrell (2012)	18–30	(56.0)	(18.8)	NR			White	(44.0)	(30.4)	≥College	(68.0)	(40.3)
Cao (2017)	≤29	393 (80.7)	241 (63.9)	Gay	373 (76.6)	257 (68.2)	NR			<College	119 (24.4)	122 (32.4)
	≥30	94 (19.3)	136 (36.1)	Other	114 (23.4)	120 (31.8)				≥College	368 (75.6)	255 (67.6)
PhillipsII (2015)	18–29	18–29	NR	Gay	1668 (83.6)	NR	White	1207 (63.7)	NR	<College	809 (40.6)	NR
				Other	327 (16.4)		Other	688 (36.3)		≥College	1186 (59.4)	
Weiss (2017)	15–29	163 (47.2)	NR	NR			NR			NR		
	≥30	182 (52.8)										

*Abbreviations*: *SD* Standard Deviation; <College, Less than college; ≥College, College or above; *Mdn* Median age, *NR* not reported

### App usage and sexual behaviors among app-users

App usage and sexual behavior is summarized in Table 2. Among app-users, Muessig and LeGrand found 50% reported using the phone to find sexual partners [44, 45]. Ko found 88% had online sex partners in the previous 3 months [30]. Winetrobe reported that the average number of Grindr-met partners in the past 1 month was 1.84 (Standard Deviation [SD] = 2.92) [5, 13]. Goedel and Duncan reported that the average number of app-met insertive and receptive anal intercourse partners was 1.46 (SD = 6.27) and 1.07 (SD = 2.45), respectively [25, 36]. The study from Tang et al.*....* found 66.7% of app-users did not ask for HIV status of the last gay app partner before meeting in person [4].

**Table 2:** The use of apps and sexual behaviors among app-users

First author (Year)	Sexual behaviors	APP users	
		N/Mean	%/SD
Goedel (2016) Duncan (2016)	App-met IAI partners, P3M	1.46	6.27
	App-met RAI partners, P3M	1.07	2.45
Rhoton (2016)	HIV status on GSN app	2.98	8.96
Ko (2016)	Had online sex partners, P3M	352	88.0
	Unprotected anal sex online sexual partners, P6M	228	64.8
	Unprotected oral sex online sexual partners, P6M	325	88.8
Yeo (2016)	Sexual partnering via apps		
	0	86	40.4
	1–3	91	42.7
	> 3	36	16.9
Winetrobe (2014) Rice (2012)	Number of Grindr-met partners, P1M	1.84	2.92
	Ever had sex with a partner met on Grindr	147	75.4
Tang (2016)	Number of sex partners found through gay app, P6M		
	1–6	680	82.5
	> 6	144	17.5
	Number of IAI with partners met through gay app, P6M		
	0–5	629	76.3
	> 6	195	23.7
	Condomless anal sex with the last partner met through gay app	338	41.0
	Not asked for HIV status of the last gay app partner before met in person	550	66.7
Muessig (2013) LeGrand (2014)	Use phone to find sex partners	11	50.0
Chow (2016) Chow (2017)	Meeting partners via mobile apps	723	55.0
Grosskopf (2014)	Sex with a man met on the app	35	97.9
	UAI with a man met on the app	22	66.7
	Only oral or manual sex with a man met on the app	11	47.8
Cao (2017)	No. of sex partners found through the platform, P6M		
	Single	151	31.0
	Multiple	336	69.0

*Abbreviations*: *IAI* Insertive anal intercourse, *RAI* Receptive anal intercourse, *GSN* Geosocial networking, *P1M* In the past 1 month, *P3M* In the past 3 months, *P6M* In the past 6 months, *UAI* Unprotected anal intercourse

### Substance use

Two studies reported prevalence of recreational drug use among app-users (16.9% and 50.2%) [23, 34]. Two studies reported prevalence of injectable drug use (2.1% and 5.4%) [23, 31]. Goedel et al found 38.6% app-users reported having had five or more drinks containing alcohol in the previous 3 months [18]. Phillips et al reported that the prevalence of recreational drug and injectable drug use among non-users was 43.1% and 4.1%, respectively [23].

### HIV testing

The lifetime rate of HIV testing among app-users ranged from 49.1 to 96.7% [5, 23, 26, 33, 40], and ranged from 50.1 to 97.1% among non-users [23, 33, 40]. The rate of HIV testing in the preceding 12 months among app-users ranged from 10.8 to 83.2% [5, 23, 26, 27, 38], and ranged from 37.4 to 58.0% among non-users [23, 27].

### HIV prevalence

HIV prevalence was reported in sixteen studies among app-users. The range was 2.2% to 37.7%. The pooled prevalence was 6.0% (95% CI 4.0–11.0%, I^2^ = 97%, P_heterogeneity_ < 0.01), but with high heterogeneity (Additional file 2: Figure S1).

### Comparisons of ever being diagnosed with HIV/STIs between app-users and non-users

**Fig. 2:**
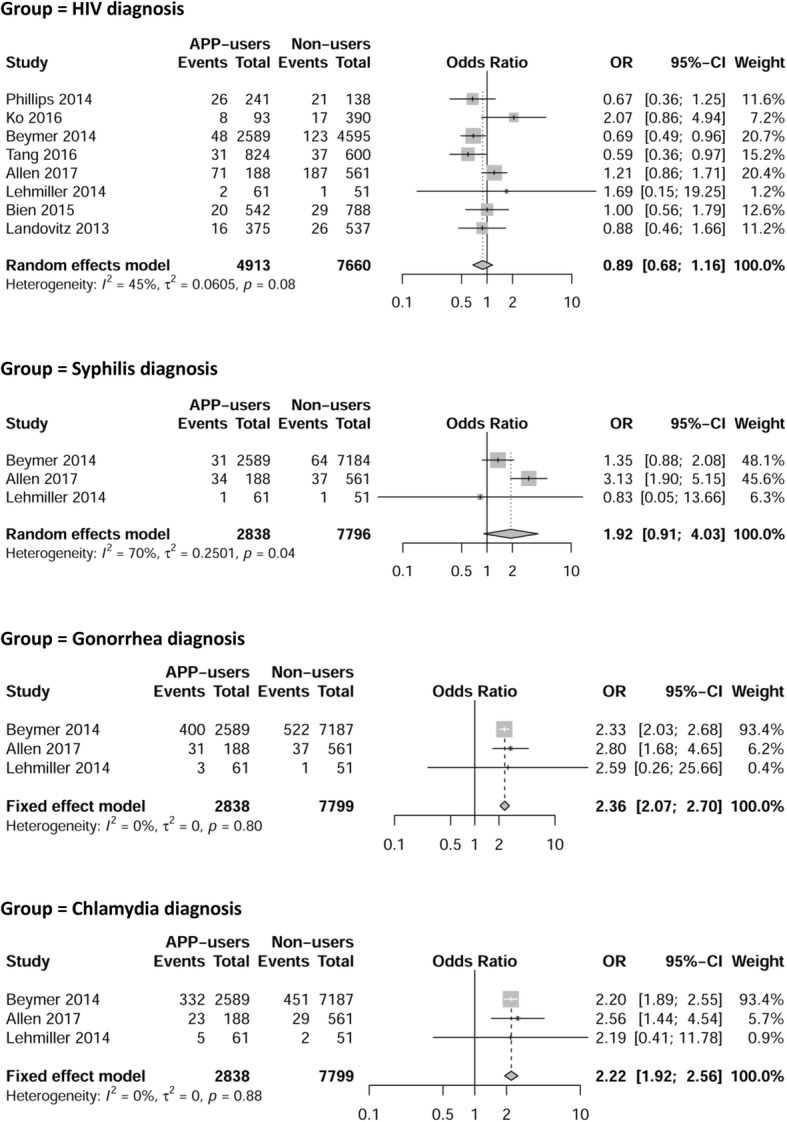
Forest plots of HIV/STI diagnosis by app-users versus non-users. Squares indicate odds ratio in each study; square size is proportional to the weight of the corresponding study in the meta-analysis; the length of the horizontal lines represents the 95% confidence interval; the diamond indicates the pooled odds ratio and 95% confidence interval

Eight studies assessed self-reported HIV diagnosis [3, 4, 6, 23, 27, 32, 33, 38]. Since we found medium heterogeneity (I^2^ = 45%, P_heterogeneity_ = 0.08) among these studies, a random-effects model was used to pool the OR. The pooled OR of 0.89 (95% CI, 0.68–1.16) for HIV diagnosis suggested no significant difference in HIV infection between app-users and non-users (Fig. 2). We applied a sensitivity analysis to explore the factors contributing to heterogeneity. Sensitivity analysis showed omitting one study in each group did not substantially change the pooled OR.

For self-reported syphilis diagnosis, we first used a fixed-effect model to pool the available data [3, 6, 32], We found that app-users were more likely to have syphilis (OR = 1.88; 95% CI, 1.37–2.59). However, we detected medium heterogeneity (I^2^ = 70%, P_heterogeneity_ = 0.04) among these studies. Therefore, we employed a random-effects model to calculate the pooled OR and found that there was no significant difference between apps-users and non-users (OR = 1.92; 95% CI, 0.91–4.03) (Fig. 2). We found that Beymer et al. contributed substantially to heterogeneity according to the results of sensitivity analysis. When this study was omitted, the pooled OR for syphilis infection became 3.00 (95% CI, 1.84–4.91, I^2^ = 0%, P_heterogeneity_ = 0.36) suggesting app-users were more likely to report syphilis infection.

Three studies assessed self-reported gonorrhea and chlamydia diagnoses [3, 6, 32]. As there was no heterogeneity for either gonorrhea (I^2^ = 0%, P_heterogeneity_ = 0.80) or chlamydia (I^2^ = 0%, P_heterogeneity_ = 0.88) diagnoses, we employed a fixed-effect model to pool the OR. The pooled OR showed app-users were more likely to report gonorrhea (OR = 2.36; 95% CI, 2.07–2.70) (Fig. 2) and chlamydia (OR = 2.22; 95% CI, 1.92–2.56) (Fig. 2) infections.

### Publication bias

We found no publication bias for these analyses by Begg’s test (all *P* > 0.05) or Egger’s test (all *P* > 0.05).

## Discussion

This was a quantitative study estimating the prevalence of HIV infection among app-users and non-users, and comparing self-reported STIs diagnoses between the two groups. In addition, we investigated app use and sexual behaviors of app-users. The prevalence of HIV infection, substance use, and HIV testing varied widely among app-users and non-users. We found that app-users engaged in several unsafe sexual behaviors. Our meta-analysis demonstrated that app-users were more likely to have syphilis, gonorrhea and chlamydia diagnosis than were non-users.

Since the early 2000s, researchers have noted that partnerships pursued through online interactions were different from venue-based interactions. Advances in communication technology may affect the sexual partnership [14]. Many studies have reported that because apps provide MSM with more efficient ways to seek sexual partners compared with other methods, apps were more likely to facilitate higher risk sexual behaviors [35, 38]. Our study was not exceptional for finding that risky sexual behaviors (e.g., greater number of sexual partners, unprotected sex) were common among app-users. Apps potentially foster risky behavior because users could carry their smart phones with them at all times. A notable finding was that app-users were more inclined to be diagnosed with STIs than were non-users. Higher prevalence of risky sexual behaviors and higher risk of STIs infections may put app-users at greater risk for HIV transmission. However, we found that the two groups were similar with respect to reported HIV diagnosis. Ko et al. found that HIV-positive MSM were more likely to use apps to seek partners. HIV-positive MSM were concerned with maintaining his attractiveness in apps, and therefore implied his serostatus in his app profile. Therefore, apps might provide these men easier channels to hide HIV positive status [27]. Taken together, the advancements in apps and the increase in MSM using these apps may produce more adverse effects on sexual health. The data demonstrate the need for increased app-based prevention interventions among MSM.

Our study found high prevalence of recreational drug use among app-users. Substance use and misuse are prevalent among MSM [46], especially alcohol and recreational drugs [47]. The National HIV Behavioral Surveillance showed that 42% of MSM used substances recreationally [48]. It has been reported that the use of substances was associated with HIV-related sexual risk behaviors [25, 46]. Therefore, substance use may a strong predictor of sexual risk behaviors.

We found a high rate of lifetime HIV testing among both app-users and non-users, and a slightly higher rate of HIV testing in the previous 12 months among app-users compared with non-users. It appears that app-users may be likely to utilize health resources, because MSM engaging in risky behaviors may recognize the need for HIV testing [49]. As has been validated by several studies, app-users were more likely to engage in unsafe sex [7–9]. Therefore, for app-users, frequent testing might be associated with high-risk sexual behaviors. Nevertheless, we cannot verify this association in the present study. In fact, several studies reported that many app-users never underwent HIV testing [33, 40]. A study conducted in Peru reported that 60% of MSM with newly diagnosed HIV infection had not been tested within 12 months [49], suggesting that non-testers might be at high risk for infection. This is a significant issue, because infected non-testers can unknowingly transmit HIV to their partners [50, 51], resulting in an increasing rate of HIV infection. This suggests that, integrating HIV testing into routine medical care might increase testing in high-risk MSM.

Our study had a few limitations. First, most studies were descriptive, without a comparable group (referred to non-users). This presented an obstacle for making comparisons between app-users and non-users. Second, the association between app use and sexual risk behaviors/STIs may not imply a causal relation.

## Conclusions

Increased app use among MSM has been linked to casual sexual partners and unsafe sex. We suspect that app based dating offers avenues for more discreet dating that offers a possibility of increases in STIs. This situation has important implications for HIV prevention. Our analyses support the notion that MSM who seek sexual partners using apps may be more likely to have STIs infections than are non-users. As smartphone use increases, acceptable mobile platforms for HIV prevention are recommended. In addition, more studies, especially longitudinal studies, are needed to confirm the relative risk between app-user and non-user..

## Additional files

Additional file 1: **Table S1.** Characteristics of studies included in the meta-analysis. (DOCX 26 kb)

Additional file 2: **Figure S1.** Forest plots of HIV diagnosis among app-users. Proportion refers to the rate of HIV diagnosis among app-users; squares indicate proportion in each study; square size is proportional to the weight of the corresponding study in the meta-analysis; the length of the horizontal lines represents the 95% confidence interval; the diamond indicates the pooled proportion and 95% confidence interval. (TIF 275 kb)

## Abbreviations

App: Geosocial networking smartphone application
App-users: App-using MSM
CI: Confidence interval
GPS: Global positioning system
HIV: Human immunodeficiency virus
MSM: Men who have sex with men
Non-users: MSM who used different channels to seek sex partners
OR: Odds ratio
SD: Standard Deviation
STI: Sexually transmitted infection
