# Supplementary material for: The use of geosocial networking smartphone applications and the risk of sexually transmitted infections among men who have sex with men: a systematic review and meta-analysis
Source: BMC Public Health. 2018 Oct 16;18:1178. doi: 10.1186/s12889-018-6092-3 (PMC6192100; doi:10.1186/s12889-018-6092-3)
Supplement: Supplementary file 1 — Table S1. Characteristics of studies included in the meta-analysis. (DOCX 26 kb) [file 12889_2018_6092_MOESM1_ESM.docx]

**Supplementary Table 1 Characteristics of studies included in the meta-analysis**

| First author (Year) | Study location | Study design | Study period | Recruitment method | Main study objective |
| --- | --- | --- | --- | --- | --- |
| Goedel (2015) | Atlanta, USA | Cross-sectional study | Jan, 2015 | Participants were recruited through advertisements on Grindr | To study GSN app use and sexual behaviors of MSM |
| Goedel (2016) Duncan (2016) | New York, USA | Cross-sectional study | Mar, 2015 | Participants were recruited through broadcast advertisements on Grindr | To study the associations between app use and condom-less sexual behaviors among MSM who use GSN apps |
| Phillips (2014) | Washington, USA | Cross-sectional study | Jul-Dec, 2011 | Participants were recruited through Venue-based sampling | To study GSN app use and sex-seeking behaviors of MSM |
| Rhoton (2016) | Maharashtra, India | Cross-sectional study | Sept, 2013-May, 2014 | Participants were recruited through banner advertisements on gay websites, social media advertisements and posts | To describe how Indian MSM self-presentation on GSN apps contributes to sexual preferences, HIV or STI disclosure |
| Holloway (2015)  Holloway (2015) | Los Angeles, USA | Cross-sectional study | Aug 8-Oct 3, 2011, Dec 5, 2011-Jan 3, 2012 | Participants were recruited through profiles of research assistants on a GSN Apps | To study characteristics in MSM who used GSN apps |
| Ko (2016) | Taiwan, China | Cross-sectional study | Jan 12-Feb 13, 2013 | Participants were recruited through chat rooms, gay-related websites, and profile pages | To study the sexual and substance-use behaviors of MSM who use GSN apps |
| Beymer (2014) | Los Angeles, USA | Cross-sectional study | Aug, 2011-Jan, 2013 | Data were collected electronically in face-to-face interviews | To study characteristics in MSM who used GSN apps vs in person or via internet social networking methods to meet other men |
| Beymer (2016) | Los Angeles, USA | Cross-sectional study | Nov 24, 2013-May 19, 2014 | Participants were recruited from Amazon’s Mechanical Turk (an online survey engine) platform | To study the associations between apps use and health-related behaviors |
| Yeo (2016) | Hong Kong, China | Cross-sectional study | Nov, 2014-Feb, 2015 | Participants were recruited through gay apps, a gay online forum, the Hong Kong Pride Parade, gay bars, and social service organizations serving MSM | To study the sexual risk behaviors of MSM who using GSN apps |
| Winetrobe (2014)  Rice (2012) | Los Angeles, USA | Cross-sectional study | Aug 8, 2011 - Oct 3, 2011 | Participants were recruited through an anonymous online questionnaire survey | To study characteristics in MSM who used GSN apps |
| Tang (2016) | China | Cross-sectional study | Oct, 2014 | Participants were recruited through Chinese gay websites | To study characteristics in MSM who used GSN apps |
| Muessig (2013)  LeGrand (2014) | North Carolina, USA | Cross-sectional study | Dec 2011-Jan 2012 | Study advertisements was posted in an infectious disease clinic, health departments and other testing facilities, libraries, gyms, and websites frequented by black MSM | To understand needs in health messaging in young black MSM |
| Chow (2017)  Chow (2016) | Melbourne, Australia | Cross-sectional study | Jul 13, 2014 - Jun 30 , 2015 | Participants were recruited through questionnaire-based study that was conducted at the Melbourne Sexual Health Centre | To study characteristics in MSM who used GSN apps vs other sources for meeting partners |
| Allen (2017) | Chicago, Kansas City, Fort Lauderdale, USA | Cross-sectional study | Jun - Oct 2014 | Participants were recruited through convenience sampling with online and print advertisements/flyers, referrals, and venue-based outreach in 3 US city-Metropolitan Statistical Areas | To examine the association of self-reported STIs and use of mobile phones |
| Bien (2015) | China | Cross-sectional study | May, 2013 | Participants were recruited through two large MSM website | To investigate the sexual risk profiles of gay app users |
| Rendina (2013) | New York , USA | Cross-sectional study | Dec, 2012 | Participants were recruited through Advertisements on Grindr | To study HIV testing in MSM who used Grindr |
| Grosskopf (2014) | New York , USA | Cross-sectional study | Nov 2009 - Feb 2011 | Participants were recruited using both passive and active online/offline methods | To study characteristics in MSM who used GSN apps vs Internet websites |
| Goedel (2016) | New York , USA | Cross-sectional study | Mar, 2015 | Participants were recruited through broadcast advertisements on GSN apps | To study characteristics in MSM who used GSN apps |
| Lehmiller (2014) | USA | Cross-sectional study | NR | Participants were recruited through solicitation notices posted on various Facebook and Twitter feeds for sexuality interest groups and LGBT student center listservs at US colleges and universities | To study characteristics in men who used GSN apps vs other sources to meet other men |
| Goedel (2017) | Los Angeles, USA | Cross-sectional study | Oct 2010 - Mar 2011 | Participants were recruited through computer assisted self-interview based survey | To study the characteristics of YMSM who use Grindr |
| Landovitz (2013) | Los Angeles, USA | Cross-sectional study | Oct 01, 2010 - Mar 31, 2011 | Study profile on Grindr was created to recruit MSM at venues, bars, dance clubs, commercial sex venues, parks | To study characteristics in MSM who used Grindr and feasibility of using Grindr to engage MSM in HIV prevention studies |
| Burrell (2012) | Los Angeles, USA | Cross-sectional study | May 10, 2010 - Jul 7, 2010 | Broadcast ad shown to Grindr users in LA. Questionnaire was completed via phone. CASI questionnaire was completed during clinic visits | To study acceptability of carrier methods for rectal microbicides in men and women with RAI experience |
| Cao (2017) | China | Cross-sectional study | Sept-Oct, 2014 | Advertisements for study participation were put on 3 large gay Web portals | To examine the characteristics of MSM and the risks associated with seeking sex through websites, gay apps, and both platforms in China |
| PhillipsⅡ (2015) | Washington DC, USA | Cross-sectional study | Nov, 2014-Feb 2015 | Participants were recruited through banner and pop-up advertisements placed on a GSN mobile phone application | To study characteristics in men who used GSN apps |
| Weiss (2017) | Bangkok, Thailand | Cross-sectional study | Nov, 2014 | App-using MSM were recruited to an uncompensated survey through clicking on a push message placed on an app popular | To describe reported HIV and STI testing patterns of MSM app users, and highlight characteristics associated with never testing |

**Abbreviations:** GSN, geosocial-networking; app, application; NR, not reported; MSM, men who have sex with men; YMSM, young men who have sex with men
