# Supplementary figures and images for: The use of geosocial networking smartphone applications and the risk of sexually transmitted infections among men who have sex with men: a systematic review and meta-analysis
Source: BMC Public Health. 2018 Oct 16;18:1178. doi: 10.1186/s12889-018-6092-3 (PMC6192100; doi:10.1186/s12889-018-6092-3)

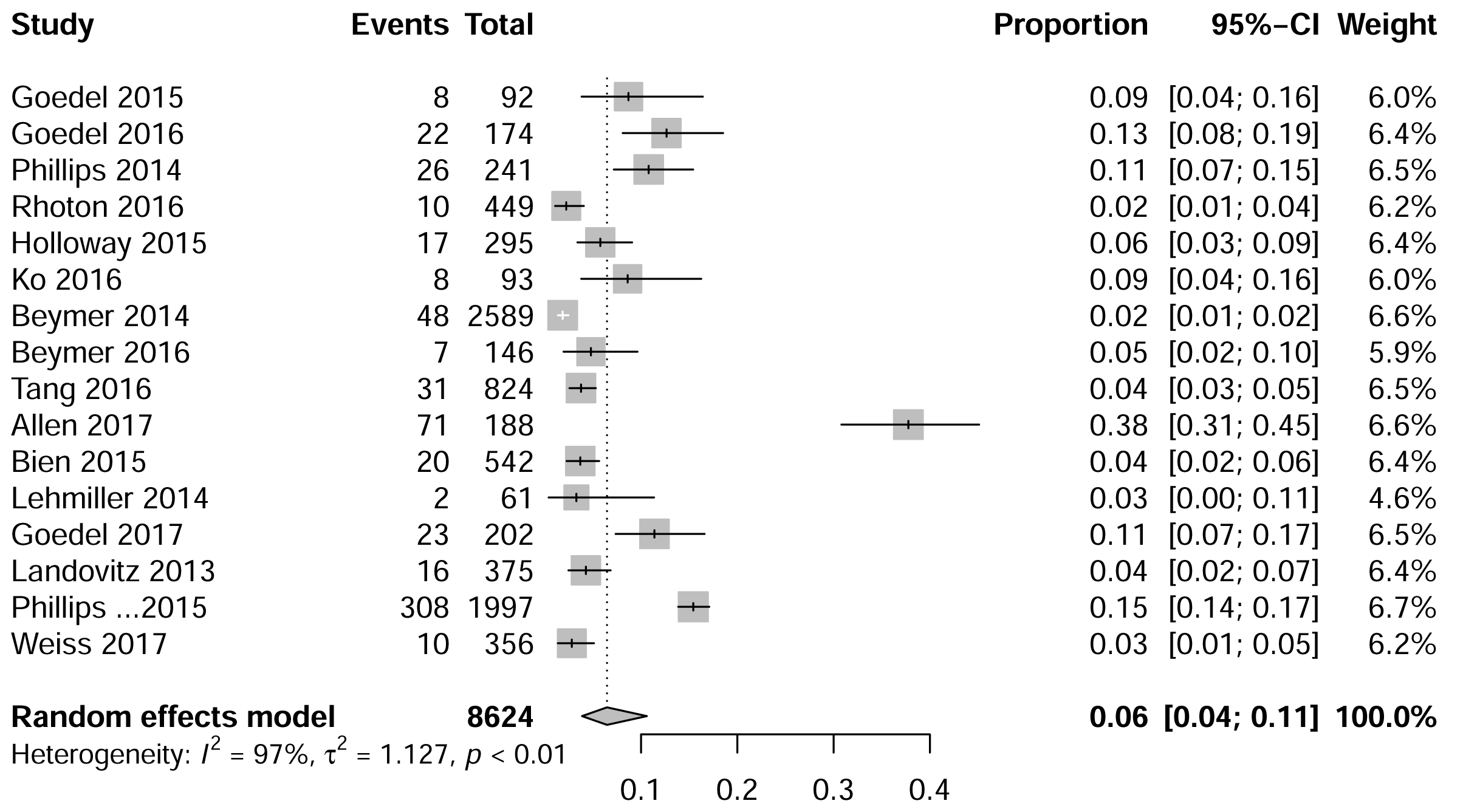

Supplement: Supplementary file 2 — Figure S1. Forest plots of HIV diagnosis among app-users. Proportion refers to the rate of HIV diagnosis among app-users; squares indicate proportion in each study; square size is proportional to the weight of the corresponding study in the meta-analysis; the length of the horizontal lines represents the 95% confidence interval; the diamond indicates the pooled proportion and 95% confidence interval. (TIF 275 kb) [file 12889_2018_6092_MOESM2_ESM.tif]
